# Exploring Sintered Fe-(Ce, Nd)-B with High Degree of Cerium Substitution as Potential Gap Magnet

**DOI:** 10.3390/ma17133110

**Published:** 2024-06-25

**Authors:** Dagmar Goll, Ralf Loeffler, Marius Boettle, Joerg Buschbeck, Gerhard Schneider

**Affiliations:** 1 Materials Research Institute, Aalen University, 73430 Aalen, Germany; ralf.loeffler@hs-aalen.de (R.L.); marius.boettle@hs-aalen.de (M.B.); gerhard.schneider@hs-aalen.de (G.S.); 2 Siemens AG, Vogelweiherstr. 1-15, 90441 Nuremberg, Germany; joerg.buschbeck@siemens.com

**Keywords:** permanent magnet, sintered magnet, rare-earth-based magnet, Ce_2_Fe_14_B, Nd_2_Fe_14_B, NdFeB, cerium, Ce substitution, coercivity

## Abstract

The more effective use of readily available Ce in FeNdB sintered magnets is an important step towards more resource-efficient, sustainable, and cost-effective permanent magnets. These magnets have the potential to bridge the gap between high-performance FeNdB and hard ferrite magnets. However, for higher degrees of cerium substitution (>25%), the magnetic properties deteriorate due to the lower intrinsic magnetic properties of Fe_14_Ce_2_B and the formation of the Laves phase Fe_2_Ce in the grain boundaries. In this paper, sintered magnets with the composition Fe70.9-(Ce_x_Nd_1-x_)18.8-B5.8-M4.5 (M = Co, Ti, Al, Ga, and Cu; with Ti, Al, Ga, and Cu less than 2.0 at% in total and Co_bal_; *x* = 0.5 and 0.75) were fabricated and analyzed. It was possible to obtain coercive fields for higher degrees of Ce substitution, which previous commercially available magnets have only shown for significantly lower degrees of Ce substitution. For *x* = 0.5, coercivity, remanence, and maximum energy product of *µ*_0_*H*_c_ = 1.29 T (*H*_c_ = 1026 kA/m), *J*_r_ = 1.02 T, and (*BH*)_max_ = 176.5 kJ/m^3^ were achieved at room temperature for *x* = 0.75 *µ*_0_*H*_c_ = 0.72 T (*H*_c_ = 573 kA/m), *J*_r_ = 0.80 T, and (*BH*)_max_ = 114.5 kJ/m^3^, respectively.

## 1. Introduction

The strongest permanent magnets today are the rare earth magnets based on FeNdB with maximum energy density (*BH*)_max_ values of 240 to 415 kJ/m^3^ at room temperature (RT). Due to the rare earth metals they contain, they are expensive, and their sustainability is compromised. Classic hard ferrite permanent magnets, on the other hand, are significantly weaker with (*BH*)_max_ values of up to 33 kJ/m^3^, but they are also significantly cheaper. Therefore, there exists a large gap between ferrite and FeNdB magnets. 

The development of permanent magnets that contain no or only very small amounts of critical rare earth (RE) elements and close the current performance gap between hard ferrite and FeNdB is becoming increasingly important. Hard magnetic phases such as Mn-based MnAl(C), Mn_2_Ga and MnBi [1,2], L_10_-FeNi [3,4,5], α-Fe_16_N_2_ [2,6], or phases with ThMn_12_ structure such as NdFe_12_(N_x_) [7] or Sm(Fe,V)_12_ [8,9] can serve as the technical basis for such gap magnets. The prerequisite for such gap magnets is sufficiently good intrinsic magnetic properties, in particular anisotropy constant *K*_1_ and saturation polarization *J*_s_. These are included in the *Q*-parameter and *κ*-parameter, which both serve as a measure of the magnetic hardness of permanent magnets and thus their suitability as permanent magnets. The well-established *Q*-parameter *Q* = 2*µ*_0_*K*_1_/*J*_s_^2^ allows for differentiating between hard (*Q* > 1) and soft (*Q* < 1) magnetic materials [10]. Alternatively, the *κ* -parameter *κ* = (*µ*_0_*K*_1_/*J*_s_^2^)^0.5^ can be used [11]. Here, *κ* > 1 is an empirical rule of thumb for a material resisting self-demagnetization. However, the challenge is that magnets can be made from those magnetic phases in bulk form and have sufficiently large coercivities *H*_c_. The larger the coercivity of a magnet at RT, the larger the coercivity at higher temperatures. Together with the Curie temperature *T*_C_, this determines the maximum operating temperature of the magnet. For example, values of *µ*_0_*H*_c_ = 0.34 T and (*BH*)_max_ = 67 kJ/m^3^ [12] were achieved for a MnBi sintered magnet, and values of µ_0_*H*_c_ = 0.32 T and (*BH*)_max_ = 56 kJ/m^3^ for a MnAl(C) hot-extruded magnet [13]. 

Alternatively, the better and more effective use of the RE element cerium (Ce) is a possible approach. Fe_14_Ce_2_B also fulfills the *Q* and *κ* criteria well (Fe_14_Ce_2_B: *Q* = 2.64, *κ* = 1.15; Fe_14_Nd_2_B: *Q* = 4.2, *κ* = 1.44). From a strategic point of view, the partial substitution of the more critical neodymium (Nd) with Ce in FeNdB permanent magnets (with as much Ce as possible) is therefore interesting in terms of resource and economic efficiency and sustainability [14,15]. This applies equally to all types (sintered [14,15,16], melt-spun [15,17,18,19], hot-deformed [15,20,21,22,23]) of today’s most powerful FeNdB magnets. However, there are significant differences between the Fe-Ce-B system and the Fe-Nd-B system, particularly with regard to intrinsic magnetic properties of the hard magnetic 14:2:1 phase, phase equilibria, and the resulting microstructures and magnetic properties. The hard magnetic Fe_14_Ce_2_B phase, for example, has lower intrinsic magnetic property values such as saturation polarization *J*_s_, anisotropy constant *K*_1_, and Curie temperature *T*_C_ (Fe_14_Ce_2_B: *J*_s_ = 1.17 T, *K*_1_ = 1.44 MJ/m^3^, *T*_C_ = 151 °C; Fe_14_Nd_2_B: *J*_s_ = 1.61 T, *K*_1_ = 4.3 MJ/m^3^, *T*_C_ = 312 °C) [24]. Furthermore, Fe-Ce-B magnets are composed of hard magnetic Fe_14_Ce_2_B, Laves Fe_2_Ce, and nonmagnetic impurity Fe_2_CeB_2_ phases, whereas Fe-Nd-B magnets contain hard magnetic Fe_14_Nd_2_B, as well as paramagnetic Nd-rich and nonmagnetic impurity Fe_4_Nd_1.1_B_4_ phases [14]. The Laves phase Fe_2_Ce is a peritectic reaction product at 925 °C, while the Nd-rich phase is a eutectic reaction product at 655 °C [14]. Nevertheless, Fe_2_Ce is assumed to have the same effect as the Nd-rich phase in FeNdB sintered magnets. However, this is still disputed [23]. Fe_2_Ce is paramagnetic at room temperature (*T*_C_ = −43 °C) and liquid at sintering temperature [25]. This liquid phase wets the 14:2:1 grains and solidifies to Fe_2_Ce, which leads to magnetic decoupling of the grains [26]. The Fe_2_Ce phase tends to aggregate at triple junctions of 14:2:1 grains [27,28]. With a higher electrode potential than RE-rich phase, it can also stabilize the intergranular regions and therefore may improve the corrosion resistance of magnets [23,29]. Up to 30% degree of Ce substitution, no significant amounts of the Fe_2_Ce phase occur [14,15,30]. For higher degrees of Ce substitution, Fe_2_Ce occurs in larger quantities, and the magnetic properties become significantly worse. It is expected that Fe_2_Ce acts as a potential nucleation center during magnetization reversal, thus diminishing the coercivity of the magnets [31]. When Ce-based strip-cast flakes are used at much higher Ce contents (>80%), the soft magnetic α-Fe phase is precipitated, and the hard magnetic properties of Ce-based sintered magnets are deteriorated significantly [32,33]. All this may be the reason why sintered Fe-(Nd, Ce)-B magnets with a high degree of Ce substitution (sometimes with addition of lanthanum) have only rarely been investigated [26,34,35,36,37]. For example, coercivities between *H*_c_ = 724 kA/m or *µ*_0_*H*_c_ = 0.91 T (45 at% degree of Ce substitution) and 143 kA/m or 0.18 T (86 at% degree of Ce substitution) have so far been achieved in these references for higher Ce substitution degrees in sintered magnets (for further details, see also Section 4.2). The degree of substitution in focus so far has rather been less than 40% of the total rare earth content. For the investigations, the dual-alloy process from two different starting alloys (Ce-rich, Ce-poor) was predominantly used [14,15,16,38]. Furthermore, the influence of grain boundary regulation/engineering and reconstruction was investigated [39,40,41,42,43].

Replacing higher content of Nd in FeNdB with Ce is the focus of the current paper. A single-alloy sintered magnet with 50 at% Ce substitution of composition Fe70.9-(Ce_0.5_Nd_0.5_)18.8-B5.8-M4.5 (M = Co, Ti, Al, Ga, Cu) is compared to its counterpart of composition Fe70.9-(Ce_0.75_Nd_0.25_)18.8-B5.8-M4.5 (M = Co, Ti, Al, Ga, Cu) with 75 at% Ce substitution level. Both magnets were fabricated and systematically analyzed. A Ce-free sample (0 at% Ce substitution) of the same composition was also used for comparison purposes. It is shown that coercive fields and good squareness of hysteresis loops for these higher degrees of Ce substitution can be obtained, for which previous commercially available magnets have only shown for significantly lower degrees of Ce substitution (35 at%).

## 2. Materials and Methods

### 2.1. Magnet Compositions

The highly cerium-substituted chemical compositions Fe70.9-(Ce_0.5_Nd_0.5_)18.8-B5.8-M4.5 and Fe70.9-(Ce_0.75_Nd_0.25_)18.8-B5.8-M4.5 (M = Co, Ti, Al, Ga, Cu; with Ti, Al, Ga, and Cu less than 2.0 at% in total and Co_bal_) were selected for the experiments. The excess of RE compared to 14:2:1 stoichiometry, as required for sintered magnets, was 7 at%. The magnets are designated Ce0.5 and Ce0.75 in the following. For a first comparison, we used a Ce-free variant of the Ce-containing sintered magnets (nomenclature Ce0) of chemical composition Fe71.8-Nd18.4-B5.8-M4.0. It should be noted that the comparison is only possible to a limited extent, as Ce-free magnets even with the same composition may have slightly different process windows in order to obtain correspondingly optimized properties.

### 2.2. Magnet Synthesis

**Figure 1:**
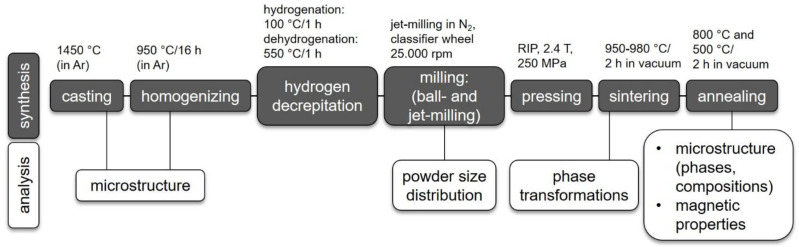
Flow chart of the synthesis procedure (including processing conditions) and analysis methods used.

Figure 1 shows the procedure for magnet synthesis schematically in the form of a flow chart. Accordingly, book-mold-cast (BMC) alloys were fabricated by induction melting at 1450 °C (VTC 200V/Ti, Indutherm GmbH, Walzbachtal, Germany ) in an Ar atmosphere from the constituent elements and a high-purity FeB pre-alloy (>99.9%) (Less Common Metals Ltd., Ellesmere Port, United Kingdom; Thermo Fisher Scientific Inc., Waltham, MA, USA; MaTeck GmBH, Jülich, Germany). For homogenization, the BMC alloys were heat-treated at 950 °C for 16 h in an Ar atmosphere. For magnet manufacturing, a research pilot line was used. The heat-treated alloys were hydrogen decrepitated (hydrogenation for 1 h at 100 °C, partly dehydrogenation for 1 h at 550 °C). For pre-crushing, ball milling (5 min) was applied.

**Figure 2:**
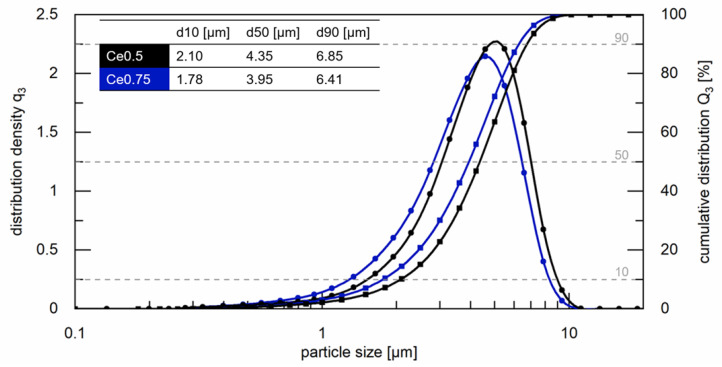
Particle size distributions using laser diffraction of the powders used for fabrication of the Ce0.5 and Ce0.75 sintered magnets. The values of *d10*, *d50,* and *d90* are also listed.

**Figure 3:**
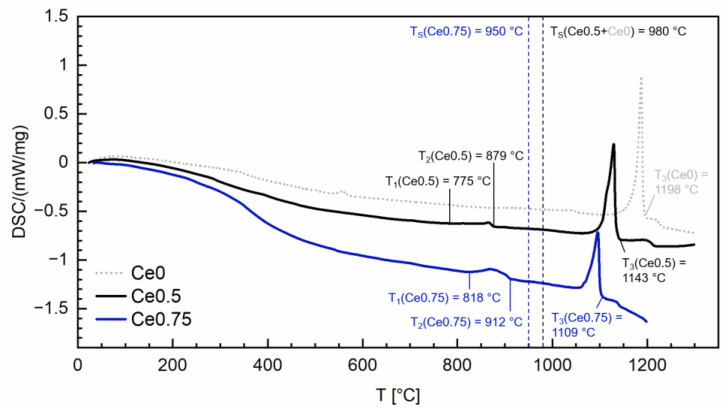
Differential scanning calorimetry (DSC) analysis performed on the Ce0.5 and Ce0.75 sintered magnets, heated from 25 °C to 1300 °C and 1200 °C with 10 °C/min, respectively. In the temperature range (*T*_1_, *T*_2_), partial melting occurs, while at (*T*_3_), the 14:2:1 phase is fully liquid. Sintering temperatures are labelled (*T*_sin_). The measurement curve for Ce0 is also shown. Here, partial melting starts at temperatures of approximately 550 °C.

Then, jet-milling in N_2_ atmosphere was used (classifier wheel 25.000 rpm) to obtain fine powders. The particle size distribution of the powders was analyzed using laser diffraction (HELOS BR, Sympatec, Clausthal-Zellerfeld, Germany). For the measurement, a lens system with a measuring range from 0.1 to 35 µm was used. Figure 2 shows the results for the two different compositions. The value d50 amounts to ≈4 µm in both cases. The powder was aligned and pressed in a magnetic field (2.4 T) using rubber isostatic pressing. The obtained green bodies were sintered in an elevator furnace at *T*_sin_ = 950 °C (Ce0.75) and 980 °C (Ce0.5) for 2 h (Ce0: *T*_sin_ = 980 °C). Finally, the sintered magnets underwent two-step post-annealing in vacuum at 800 °C and 500 °C for 2 h each. Differential scanning calorimetry (DSC) analysis (DSC 404 F3 Pegasus, Netzsch, Selb, Germany) was performed on the sintered magnets to obtain information about phase formation temperatures and to fine-tune the sintering temperature *T*_sin_. Figure 3 shows the results for the two different compositions. For Ce0.5 (or Ce0.75), the temperature range where partial melting occurs is 775–879 °C (or 818–912 °C), and the hard magnetic 14:2:1 phase is liquid at 1143 °C (or 1109 °C). For comparison the measurement curve for Ce0 is also shown. Here, partial melting starts at temperatures of approximately 550 °C. 

### 2.3. Magnet Analysis

The extrinsic magnetic properties of the magnets (coercivity *H*_c_, remanence *J*_r_, maximum energy product (*BH*)_max_) and their temperature dependence were determined from hysteresis loop measurements (Quantum Design, San Diego, CA, USA PPMS-9T). The samples were saturated in the device at 9 T. The field rate for the measurements was 50 Oe/s. The accuracy of the determined *H*_c_ and *J*_r_ values was 0.5%. The temperature at which the ferromagnetic behavior disappears (Curie temperature *T*_C_ of the 14:2:1 phase) was determined from *J*-*T* kinkpoint measurements. After saturation of the sample at 9 T, the magnetic polarization *J* was measured as a function of temperature *T* (temperature step: 5 K/min, applied magnetic field during measurement: 50 Oe (heating and cooling)). The Curie temperature *T*_C_ was derived from the turning point of the cooling curve. The magnet density *ρ* was determined based on Archimedes’ principle (*ρ* (g/cm^3^): Ce0/7.52 ± 0.01, Ce0.5/7.62 ± 0.01, Ce0.75/7.61 ± 0.02) to convert the unit of magnetic polarization into tesla. The microstructure was investigated on polished microsections of the magnets. The microsections were prepared using materialographic methods and analyzed by optical microscopy (ZEISS Axio Imager.Z2m, Carl Zeiss AG, Oberkochen, Germany) and scanning electron microscopy (SEM, ZEISS Sigma 300 VP, Carl Zeiss AG, Oberkochen, Germany). In all microstructure examinations, the texture axis was in the microsection plane, and in the microstructure images, it was in the vertical direction. Optical microscopy, including quantitative microstructural analysis QMA, was applied to determine the area fractions of the respective existing phases. The microstructures were segmented by a convolutional neural network (U-Net). This was specifically trained on two microstructure images per sample. For QMA, 20 images per sample (magnification 1000×) were evaluated using this algorithm. From the segmented images, the area fractions of the existing phases were determined. SEM, including energy dispersive X-ray analysis (EDS), was applied to determine the chemical composition of the magnets and of the phases present. For EDS, the acceleration voltage used was 20 kV. The energy resolution was 120–130 eV for Mn Ka 5.89 keV. Additionally, electron backscatter diffraction (EBSD, EDAX Hikari, OIM v8.6 orientation imaging microscopy, stepsize: 330 nm, threshold: minimum 20 pixels per grain that results in an equivalent diameter of ≈1.5 µm) was applied to obtain grain size and grain orientation information. 

For the classification and evaluation of the results, a commercial sintered magnet (supplier ChenYang Technologies GmbH and Co. KG, Finsing, Germany) was used.

## 3. Results

### 3.1. Magnetic Properties

**Figure 4:**
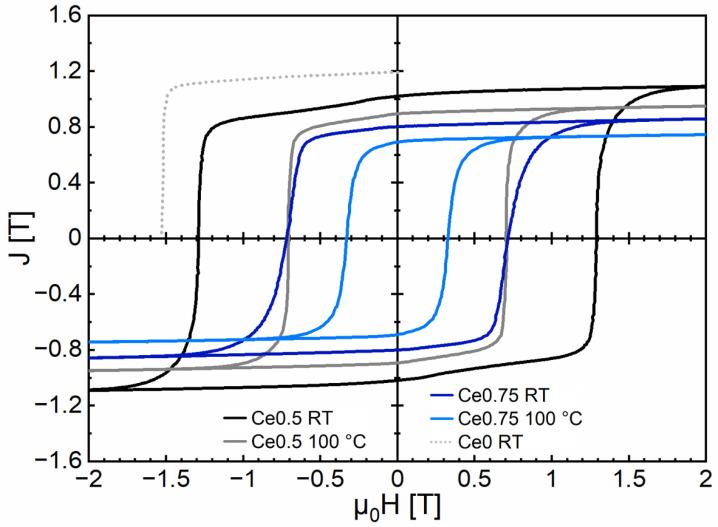
Hysteresis loops of Fe70.9-(Ce_0.5_Nd_0.5_)18.8-B5.8-M4.5 (Ce0.5) and Fe70.9-(Ce_0.75_Nd_0.25_)18.8-B5.8-M4.5 (Ce0.75) (M = Co, Ti, Al, Ga, and Cu; with Ti, Al, Ga, and Cu less than 2.0 at% in total and Co_bal_) sintered magnets at RT (20 °C) and 100 °C. Additionally, the RT curve of the Ce0 sintered magnet is shown in the second quadrant (dashed line, measurement performed in hysteresis graph after saturation at 7 T).

**Figure 5:**
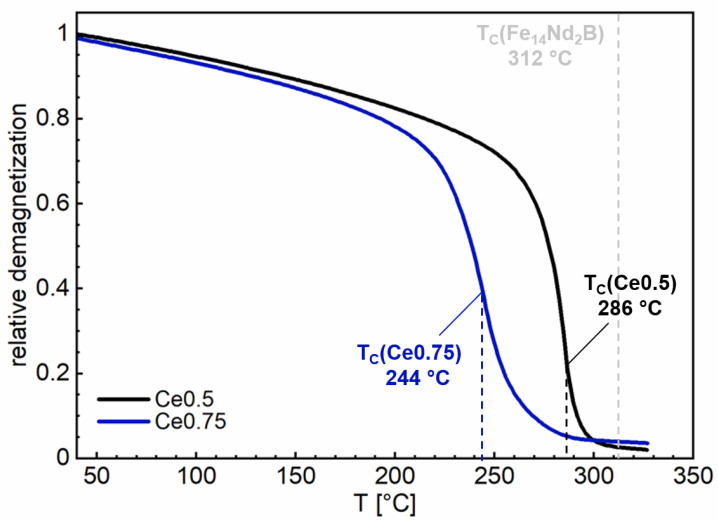
*J*-*T* kinkpoint measurements of Fe70.9-(Ce_0.5_Nd_0.5_)18.8-B5.8-M4.5 (Ce0.5) and Fe70.9-(Ce_0.75_Nd_0.25_)18.8-B5.8-M4.5 (Ce0.75) (M = Co, Ti, Al, Ga, and Cu; with Ti, Al, Ga, and Cu less than 2.0 at% in total and Co_bal_) sintered magnets. The Curie temperature *T*_C_ is derived from the turning point of the curves. The Curie temperature of Fe_14_Nd_2_B of 312 °C is also shown.

Figure 4 shows the hysteresis loops measured at room temperature (RT) (20 °C) and at 100 °C for Fe70.9-(Ce_0.5_Nd_0.5_)18.8-B5.8-M4.5 (Ce0.5) and Fe70.9-(Ce_0.75_Nd_0.25_)18.8-B5.8-M4.5 (Ce0.75) (M = Co, Ti, Al, Ga, and Cu; with Ti, Al, Ga, and Cu less than 2.0 at% in total and Co_bal_). All relevant magnetic properties are summarized in Table 1. The Ce-free sintered magnet Ce0 is included in the second quadrant for comparison. At RT, the Ce0.5 sintered magnet exhibited a coercivity of *µ*_0_*H*_c_ = 1.29 T, a remanence of *J*_r_ = 1.02 T, and a maximum energy product of (*BH*)_max_ = 176.5 kJ/m^3^. The Ce0.75 sintered magnet at RT still exhibited values of *µ*_0_*H*_c_ = 0.72 T, *J*_r_ = 0.80 T, and (*BH*)_max_ = 114.5 kJ/m^3^ for the magnetic properties. It is apparent that with increasing Ce content and increasing temperature, the magnetic properties continuously decreased. For the corresponding temperature coefficients *α* and *β* (RT/100 °C) of the remanence and the coercivity, the values *α* = 0.15 and *β* = 0.57 (Ce0.5) and α = 0.17 and *β* = 0.68 (Ce0.75) were determined. This shows that the temperature stability of the magnetic properties decreased slightly as the degree of Ce substitution increased. A similar behavior was observed for the Curie temperature *T*_C,_ which decreased from 286 °C (Ce0.5) to 244 °C (Ce0.75). The related J-T kinkpoint measurements are shown in Figure 5. For the Ce0 sintered magnet, values of *µ*_0_*H*_c_ = 1.53 T, *J*_r_ = 1.19 T, and (*BH*)_max_ = 271 kJ/m^3^ were obtained. All values were larger than for Ce0.5 and Ce0.75. The magnetic properties are discussed in more detail in Section 4 (Discussion), correlating with the microstructure and compared with previously achieved literature values.

### 3.2. Microstructure

#### 3.2.1. Phase Identification

**Figure 6:**
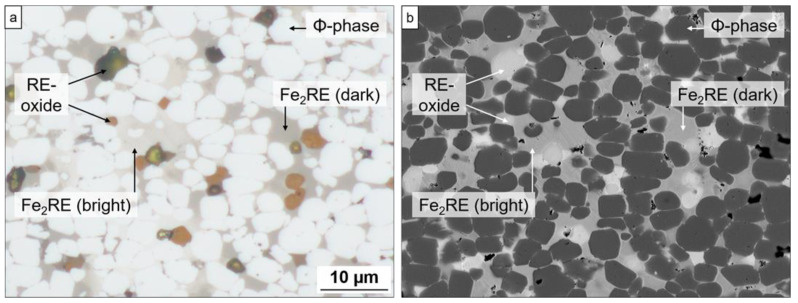
Correlative optical microscopy (**a**) with SEM-BSE and SEM-EDS analysis (**b**) used to identify the phases shown for sample Fe70.9-(Ce_0.5_Nd_0.5_)18.8-B5.8-M4.5 (Ce0.5). Measured mean composition (at%) for 14:2:1 (ϕ-phase) was Fe78.2Nd7.4Ce5.4M_bal_ and for Fe_2_RE Fe59.1Nd6.3Ce27.3M_bal_.

Both samples show a fully dense multiphase microstructure without significant pores and only minor breakouts resulting from the materialographic preparation of the microsections (grinding and polishing). This is illustrated in Figure 6, which shows representative correlative images of an identical sample area for sample Ce0.5. In the bright-field image (Figure 6a) acquired with the optical microscope, the hard magnetic 14:2:1 phase (*ϕ*) appears as rounded bright grains of light grey color, the Fe_2_RE Laves phase as bulky aggregates of pale beige to brownish color, and the RE-oxides as round grains of reddish-brown color. The corresponding SEM-BSE image (Figure 6b) was used to determine the chemical compositions of the occurring phases by EDS-analysis.

#### 3.2.2. Quantitative Analysis of Phase Content

**Figure 7:**
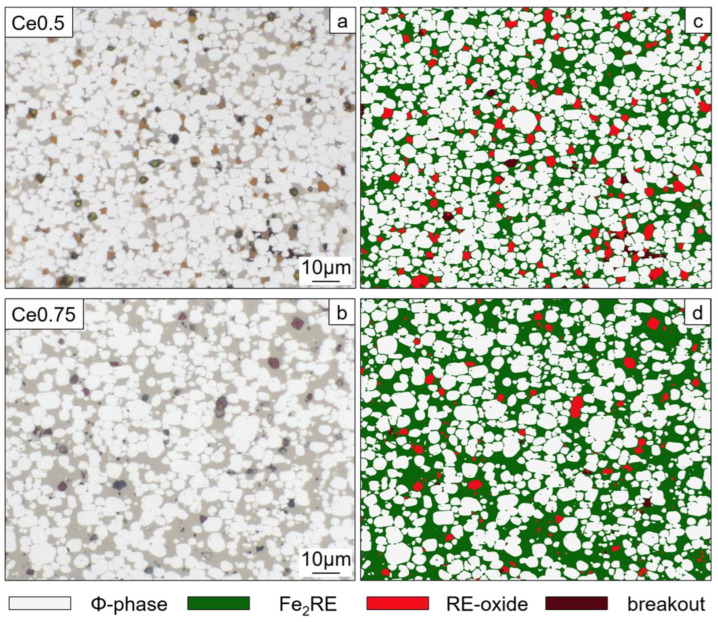
Quantitative analysis of phase content in Fe70.9-(Ce_0.5_Nd_0.5_)18.8-B5.8-M4.5 (Ce0.5) and Fe70.9-(Ce_0.75_Nd_0.25_)18.8-B5.8-M4.5 (Ce0.75) (M = Co, Ti, Al, Ga, and Cu; with Ti, Al, Ga, and Cu less than 2.0 at% in total and Co_bal_) sintered magnets. Light optical micrographs of representative bright-field views (**a**,**b**) with corresponding false color representation of detected phases (**c**,**d**).

**Figure 8:**
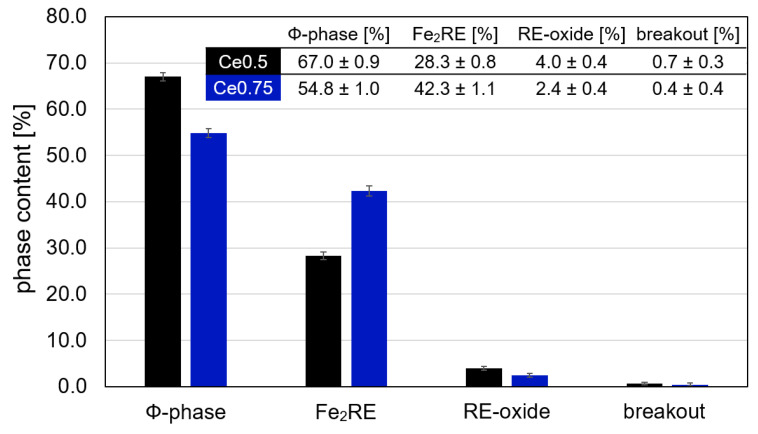
Summary of phase fractions from quantitative microstructure analysis of samples Fe70.9-(Ce_0.5_Nd_0.5_)18.8-B5.8-M4.5 (Ce0.5) and Fe70.9-(Ce_0.75_Nd_0.25_)18.8-B5.8-M4.5 (Ce0.75) (M = Co, Ti, Al, Ga, and Cu; with Ti, Al, Ga, and Cu less than 2.0 at% in total and Co_bal_).

The amount of occurring phases in both samples was determined by quantitative image analysis of optical bright field images (Figure 7). A summary of the measured phase contents is given in Figure 8. From the analysis, it became clear that in both samples, the main constituent was the hard magnetic 14:2:1 phase. However, its content was 12% higher in sample Ce0.5 compared to Ce0.75 (67.0% vs. 54.8%). The amount of Fe_2_RE Laves phase, on the other hand, was 14% less in the Ce0.5 magnet (28.3% vs. 42.3%, respectively). The two magnets contained similar amounts of RE-oxides, which was slightly higher in sample Ce0.5 (4.0% vs. 2.4%). 

#### 3.2.3. Quantitative Analysis of Grain Size and Degree of Texture

**Figure 9:**
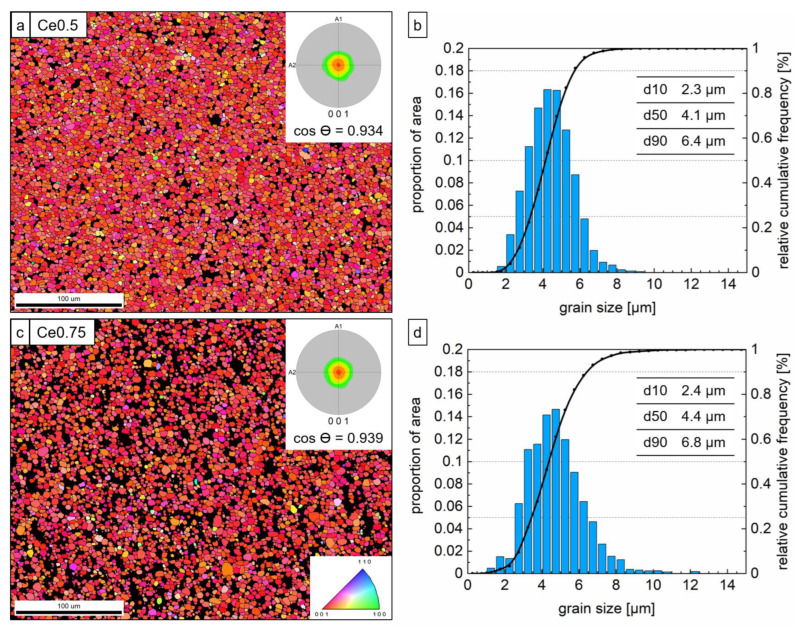
Quantitative SEM-EBSD analysis of degree of magnetic texturing (**a**,**c**) and grain size (**b**,**d**) of samples Fe70.9-(Ce_0.5_Nd_0.5_)18.8-B5.8-M4.5 (Ce0.5) and Fe70.9-(Ce_0.75_Nd_0.25_)18.8-B5.8-M4.5 (Ce0.75) (M = Co, Ti, Al, Ga, and Cu; with Ti, Al, Ga, and Cu less than 2.0 at% in total and Co_bal_).

The initial SEM-EBSD analysis of the microstructures did not appear to show major differences between the two samples regarding their grain size (Figure 9). The average grain sizes (d50) were 4.1 µm and 4.4 µm for the Ce0.5 and Ce0.75 samples, respectively. However, the analysis showed slightly coarser grains for the Ce richer Ce0.75 sample than for the Ce0.5 sample (d90 of 6.4 µm vs. 6.8 µm), ending at 14.1 µm and 9.1 µm, respectively. The comparable grain size distributions are also reflected in the comparable powder particle sizes of the two starting powders (see Figure 2). Further, the two magnets showed comparable degrees of texturing, yielding 93.4% and 93.9% for the Ce0.5 and Ce0.75 samples, respectively. This indicates that both starting powders behaved similarly during magnetic alignment in the pressing step of the green body fabrication. 

#### 3.2.4. Chemical Composition and Local Distribution of RE-Elements

For the hard magnetic 14:2:1 phase, the EDS-analysis of the Ce0.5 and Ce0.75 samples gave an average composition of Fe78.2Nd7.4Ce5.4M_bal_ and Fe78.4Nd4.2Ce8.4M_bal_, and for the Fe_2_RE Laves phase, Fe59.1Nd6.3Ce27.3M_bal_ and Fe58.7Nd3.2Ce31.2M_bal_, respectively. There appears to be a trend that the higher the Ce content of the bulk composition, the higher the Ce content in the 14:2:1 and Fe_2_RE phases. However, further evaluation shows that Nd and Ce were not distributed evenly in the different phases. Rather, they exhibited a clear preference: Nd preferentially accumulated in the hard magnetic 14:2:1 phase, while Ce concentrated in the Fe_2_RE Laves phase. This became evident when the proportions of Nd and Ce in the respective bulk compositions were compared (Table 2). For example, the 14:2:1 phase in the Ce0.5 sample contained approximately 7.8% more Nd, and the Fe_2_RE phase approximately 31.3% more Ce compared to the Nd/Ce ratio of the original starting composition (50/50). In the case of the Ce0.75 sample, the effect was slightly less but still apparent. There, the 14:2:1 phase was approximately 8.3% richer in Nd, whereas the Fe_2_RE phase was approximately 15.7% richer in Ce compared to the RE ratio of the alloy composition (25/75).

## 4. Discussion

### 4.1. Correlation between Microstructure and Magnetic Properties

The results of our analysis seem to verify the known correlation between Ce content, phase content and magnetic properties of sintered magnets with high Ce content. As the Ce content in the magnet increased, the remanence *J*_r_, the coercivity *H*_c_, and the fraction of the hard magnetic 14:2:1 phase decreased, while the fraction of Fe_2_RE increased, which is known to reduce *J*_r_ as a paramagnetic phase at room temperature. Nevertheless, the observed decrease in extrinsic magnetic properties was also consistent with the lower intrinsic properties of saturation polarization *J_s_* and magnetocrystalline anisotropy constant *K*_1_ as the Ce content increased in the 14:2:1 main phase towards the pure 14:2:1 Fe-Ce-B phase. Concerning our results, since both magnets exhibited an almost identical degree of magnetic texturing, the higher remanence *J_r_* (+27.5%) of the Ce0.5 magnet/sample relative to Ce0.75 can be attributed to both the 12% higher proportion of this 14:2:1 phase and the 76% higher Nd content therein. There might be a similar yet inverse correlation for the coercive field: the lower coercive field of the Ce-rich Ce0.75 magnet should be, at least, partly due to the significantly lower Nd content of the 14:2:1 phase in the magnet, lowering its *K*_1_. The extent to which the higher proportion of the Fe_2_RE phase in this magnet (+14%), and in general, might also contribute to a decrease in the coercive field has not yet been fully clarified at this stage. As expected, the Ce-free sintered magnet Ce0 showed higher values for coercivity and remanence due to its better intrinsic magnetic properties and the phases involved. The presence of low-melting phases at significantly lower temperatures and the absence of the Fe_2_Ce phase contribute to better wetting of the hard magnetic grains, thus positively influencing the resulting larger coercivity. 

### 4.2. Comparison with Other Sintered and Rapidly Quenched Magnets of Similar Ce Content in the Literature and with Commercial Magnets

For a better classification and evaluation of the properties achieved for the sintered magnets Ce0.5 and Ce0.75, they are compared in Table 3 with values from the literature for sintered and rapidly quenched magnets with a comparable degree of Ce substitution as well as with those of a commercial magnet. The larger the coercivity of a magnet at room temperature, the larger the coercivity at higher temperatures. This, together with the Curie temperature, determines the maximum operating temperature of the magnet.

It has to be noted that both the Ce0.5 and Ce0.75 magnets showed exceptionally high coercivity compared to other sintered magnets of similar Ce content presented in the literature [26,34,35,36]. The Ce0.5 sintered magnet had an even slightly higher coercivity (*µ*_0_*H_c_*: 1.29 T vs. 1.27 T) than a commercially available magnet with only a 34.6% Ce substitution level of the total RE content (supplier ChenYang Technologies GmbH and Co. KG, Finsing, Germany, grade N35, measured composition Fe81.3Nd6.8Pr2.4Ce5.5Gd1.2M_bal_ (M: Co, Ti, Al, Cu) in at%, analysis of chemical composition and magnetic properties of the commercial magnet was carried out according to the described procedures in Section 2). 

The higher coercivity values achieved compared to the literature values for sintered magnets are most likely due to the higher RE/TM ratio. This ensures a sufficient grain boundary phase for effective magnetic decoupling of the hard magnetic grains (despite Fe_2_Ce formation). Consequently, this also leads to slightly lower remanence and (*BH*)_max_ values compared to literature values. Furthermore, the magnets were processed using a single-alloy process (and BMC alloys), whereas the literature used a dual-alloy process (and strip-cast alloys). In the latter case, two starting alloys are used (Ce-rich, Ce-poor). Since the soft magnetic *α*-Fe phase precipitates at higher Ce contents in Ce-based strip-cast material, this could be another reason for the lower coercivities reported in the literature, especially for high Ce substitution levels (>80%) [35,36]. The addition of La modifies the stability window for the Fe_2_Ce phase and also has a favorable effect on the temperature stability of the magnetic properties. This enables higher values for the remanence and (*BH*)_max_ even at higher Ce contents such as 75% with still passable coercivities (even if smaller than without La addition) [37]. It has to be noted that the comparison with sintered magnets from the literature can only be made qualitatively. For a quantitative comparison, the grain size, the degree of texture, and the type/type/number of additives used must also be taken into account. However, these parameters are generally not fully specified in the literature.

In contrast to sintered magnets, rapidly quenched magnets showed nanocrystalline structures instead of microcrystalline ones. Additionally, they were isotropic rather than anisotropic. Consequently, the remanence was smaller by at least a factor of 2, and the maximum energy product by a factor of 4. Furthermore, the hysteresis curves had smaller squareness. Moreover, in rapidly quenched magnets, the Fe_2_Ce phase is not formed as distinctively. This explains why over-stoichiometric rapidly quenched magnets, as in [45], achieve similar coercivities but significantly smaller remanence and (*BH*)_max_ values. Due to their nanocrystalline structure, stoichiometric compositions are also possible for rapidly quenched magnets, unlike sintered magnets. However, due to the lack of a grain boundary phase, the coercivities achieved are generally smaller. If the grain size is sufficiently small (<50 nm), an increase in remanence can occur due to direct contact between the hard magnetic grains, facilitating exchange coupling between the grains [47], as observed in [46].

### 4.3. Potential as Gap Magnets

**Figure 10:**
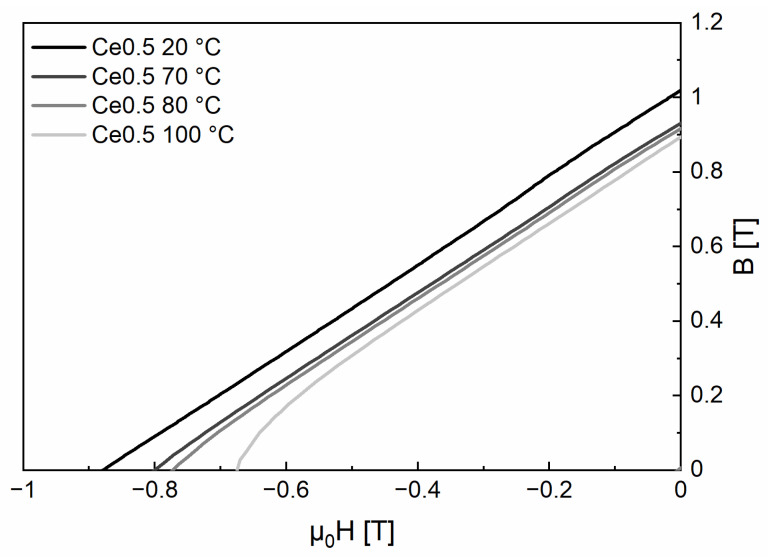
*B*-*H*-demagnetization curve in the second quadrant between the remanence *B*_r_ and the coercivity *H*_cB_ in the temperature range 20–100 °C for sample Fe70.9-(Ce_0.5_Nd_0.5_)18.8-B5.8-M4.5 (Ce0.5) (M = Co, Ti, Al, Ga, and Cu; with Ti, Al, Ga, and Cu less than 2.0 at% in total and Co_bal_). Linearity is important for the application in electrical machines.

The produced lab magnets clearly have the potential to fill the gap between commercial sintered ferrites and high-performance FeNdB magnets. According to Figure 10, a straight line of the *B*-*H*-demagnetization curve is observed in the second quadrant between the remanence *B*_r_ and the coercivity *H*_cB_ up to a temperature of 70 °C. This shows the relevance of the present investigation for future motor designs as well as the potential for further improvement. One important aspect for the generally high coercivity of the produced lab magnets could be their comparatively high amount of total RE content (18.8 at%), which, in turn, yields a sufficient amount of liquid grain boundary phase during sintering. This is further supported by the even and small grain size of both magnets, which indicates homogeneous and minimal grain growth, with both aspects vastly improving coercivity. Fe_2_RE, which is readily abundant in both magnets and is thought by researchers to act as a magnetically decoupling grain boundary phase, might play an important role in this respect. The presence of favorable sintering conditions is also expressed by smoothly shaped 14:2:1 grains with (at least on the micron-scale) well-covered surfaces, which in turn lead to smaller demagnetizing magnetic stray fields, exerting a stabilizing effect on the magnets’ demagnetization behavior. The good squareness of hysteresis loops of both samples, especially at elevated temperatures, supports this hypothesis. 

### 4.4. Further Investigations

Finally, it should be noted that the role and influence of the Fe_2_RE phase in particular on the coercivity of the two produced high Ce-content magnets requires further investigation. High_-_resolution analysis of the grain boundaries will provide further insight concerning decoupling efficiency.

## 5. Conclusions

High Ce-content Fe-Ce-Nd-B sintered magnets were successfully produced, in which 50% and 75% of the typical Nd content (by atoms) had been substituted by Ce. Despite such high level of substitution, the magnets exhibited square hysteresis loops and attractive magnetic properties, even at elevated temperatures. For the sintered magnet of composition Fe70.9-(Ce_0.5_Nd_0.5_)18.8-B5.8-M4.5, coercivity, remanence, and maximum energy product of *µ*_0_*H*_c_ = 1.29 T, *J*_r_ = 1.02 T, and (*BH*)_max_ = 176.5 kJ/m^3^ were obtained at RT, for Fe70.9-(Ce_0.75_Nd_0.25_)18.8-B5.8-M4.5 *µ*_0_*H*_c_ = 0.72 T, *J*_r_ = 0.80 T, and (*BH*)_max_ = 114.5 kJ/m^3^, respectively. 

The two high-Ce content sintered magnets showed smaller values of the magnetic properties compared to the Ce-free counterpart Fe71.8-Nd18.4-B5.8-M4.0 (*µ*_0_*H*_c_ = 1.53 T, *J*_r_ = 1.19 T and (*BH*)_max_ = 271 kJ/m^3^). However, the coercivities achieved were significantly higher compared to sintered magnets with similar Ce content presented in the literature, such as up to *µ*_0_*H*_c_ = 0.91 T for 45% Ce substitution level [34]. In the case of Fe70.9-(Ce0.5Nd0.5)18.8-B5.8-M4.5, they were even comparable to commercially available sintered magnets with substantially lower Ce substitution levels, for example, *µ*_0_*H*_c_ = 1.27 T for 34.6% Ce substitution level (supplier ChenYang Technologies GmbH and Co. KG, grade N35, measured composition Fe81.3Nd6.8Pr2.4Ce5.5Gd1.2Mbal (M: Co, Ti, Al, Cu) in at%). Melt-spun magnets of similar composition presented in the literature show similar coercivities but significantly smaller remanences and (*BH*)_max_ values due to their isotropic character, e.g., *µ*_0_*H*_c_ = 1.15 T for 51% Ce substitution level [45]. The decrease in remanence and coercivity with increasing degrees of Ce substitution correlates with the decrease in the hard magnetic 14:2:1 phase and the corresponding increase in the Fe_2_RE phase. This is also consistent with the lower intrinsic magnetic properties *J*_s_ and *K*_1_ of the Ce-substituted 14:2:1 phase compared to Fe_14_Nd_2_B. Our present investigation reveals the potential of improvement in commercial and highly Ce-substituted Fe-Ce-Nd-Fe-B magnets, which may serve as more sustainable gap magnets for a wide range of applications. High-Ce content magnets are not only cheaper in absolute terms than Ce-free or Ce-containing magnets with a much lower Ce substitution level. Despite its significantly lower energy product (*BH*)_max_, the not yet fully optimized Ce0.75 magnet is also quite close to the price/performance ratio of the commercial sintered magnet with a 34.6% Ce substitution level (if one only considers the raw material costs of the RE metals it contains as of May 2024). One possibility for further improvement is, for example, to moderately reduce the total RE content (and thus increase the proportion of the hard magnetic 14:2:1 phase) and at the same time increase the currently achieved remanence by about 6% to approximately 0.85 T. With the achieved property profile of Ce0.5, applications can be developed in particular for motors, generators, and sensors that are used at medium temperatures (up to 70 °C) and which have medium requirements for the maximum energy product. Given the significant fluctuations in raw material costs over the years, the use of more sustainable magnets becomes advantageous, especially when there are substantial cost differences between Nd and Ce due to the good availability of Ce. Therefore, it is beneficial to already have promising alternative magnets. On the other hand, gap magnets are also expected to simplify motor designs significantly, especially for servo motors (e.g., in robots), currently using hard ferrites for flux concentration.

## Figures and Tables

**Table 1:** Overview of magnetic properties of Fe70.9-(Ce_0.5_Nd_0.5_)18.8-B5.8-M4.5 (Ce0.5) and Fe70.9-(Ce_0.75_Nd_0.25_)18.8-B5.8-M4.5 (Ce0.75) (M = Co, Ti, Al, Ga, and Cu; with Ti, Al, Ga, and Cu less than 2.0 at% in total and Co_bal_) sintered magnets including coercivity *H*_C_, remanence *J*_r_, and maximum energy product (*BH*)_max_ at RT and 100 °C as well as RT/100 °C temperature coefficients *α* (remanence) and *β* (coercivity), and Curie temperature *T*_C_.

Sample	*T* (°C)	*H*_c_ (kA/m)	*µ*_0_*H*_c_ (T)	*J*_r_ (T)	*(BH)*_max_ (kJ/m^3^)	*α*	*β*	*T*_C_ (°C)
Ce0.5	RT 100	1026 561	1.29 0.70	1.02 0.89	176.5 136.9	0.15	0.57	286
Ce0.75	RT 100	573 261	0.72 0.33	0.80 0.69	114.5 65.0	0.17	0.68	244

**Table 2:** Measured compositions and proportions of Nd and Ce in the present phases relative to their respective starting alloy (baseline) in the samples Ce0.5 and Ce0.75. The proportions (+) clearly show enrichment and (-) depletion compared to their respective fraction in the original alloy composition (50/50 or 25/75). Proportions of the phases are based on EDS analysis and were calculated in relation to the total RE content of each respective phase/alloy.

Sample	RE-Element	Composition (at%)			Proportion (%)		
		Alloy (Weigh-In)	14:2:1	Fe_2_RE	Alloy (Baseline)	14:2:1	Fe_2_RE
Ce0.5	Nd	9.4	7.4	6.3	50.0	57.8 (+7.8)	18.7 (−31.3)
	Ce	9.4	5.4	27.3	50.0	42.2 (−7.8)	81.3 (+31.3)
Ce0.75	Nd	4.7	4.2	3.2	25.0	33.3 (+8.3)	9.3 (−15.7)
	Ce	14.1	8.4	31.2	75.0	66.7 (−8.3)	90.7 (+15.7)

**Table 3:** Magnetic properties (coercivity *H*_c_, remanence *J*_r_, maximum energy density *(BH)*_max_) for Ce-substituted sintered magnets and rapidly quenched magnets recently reported in the literature for comparison. Additionally, the magnetic properties of the commercial sintered magnet (supplier ChenYang) have been listed.

Chemical Composition	RE (at%)	Ce (%)	*H*_c_ (kA/m)	*µ_0_H*_c_ (T)	*J*_r_ (T)	*(BH)*_max_ (kJ/m^3^)	Ref.
**Sintered magnets**							
Commercial (supplier ChenYang)	15	35	1008	1.27	1.20	252	-
(Pr,Nd)8.4Ce5.8Fe78.4M1.4B6.1	14.2	40	660	0.83	1.25	291	[44]
(Pr,Nd)7.6Ce6.4FebalM1.0B6.0	14	45	716	0.90	1.24	292	[26]
(Pr,Nd)7.6Ce6.4FebalM1.0B6.0	14	45	724	0.91	1.21	268	[34]
(Ce,La,Nd)18.8Fe70.9B5.8M4.5	18.8	75	446	0.56	0.91	140	[37]
Ce15.5Nd2.6Fe75.5Al0.1Cu0.3B6	18.1	86	143	0.18	0.91	86	[35]
Ce13.0(Ho,Gd)2.9Fe75.0B7.0MbalCu0.23	15.9	82	133	0.17	0.80	73	[36]
**Rapidly quenched magnets**							
Nd6.89Ce7.10Fe79.98B6.03 (o.stoich.)	14	51	915	1.15	0.66	73	[45]
(Nd0.5Ce0.5)2Fe14B (stoich.)	11.76	50	493	0.62	0.73	65	[46]
(Nd0.3Ce0.7)2Fe14B (stoich.)	11.76	70	318	0.40	0.69	41	[46]

## Data Availability

The original contributions presented in the study are included in the article, further inquiries can be directed to the corresponding author.
